# Early risk factors for adult bipolar disorder in adolescents with mood disorders: a 15-year follow-up of a community sample

**DOI:** 10.1186/s12888-014-0363-z

**Published:** 2014-12-24

**Authors:** Aivar Päären, Hannes Bohman, Lars von Knorring, Gunilla Olsson, Anne-Liis von Knorring, Ulf Jonsson

**Affiliations:** Department of Neuroscience, Child and Adolescent Psychiatry, Uppsala University, Uppsala, SE-75124 Sweden; Department of Neuroscience, Psychiatry, Uppsala University, Akademiska sjukhuset, Uppsala, SE-751 85 Sweden

**Keywords:** Adolescent mood disorders, Bipolar disorder, Predictors, Long-term follow-up assessment

## Abstract

**Background:**

We aimed to outline the early risk factors for adult bipolar disorder (BPD) in adolescents with mood disorders.

**Methods:**

Adolescents (16–17 years old) with mood disorders (n = 287; 90 participants with hypomania spectrum episodes and 197 with major depressive disorder [MDD]) were identified from a community sample. Fifteen years later (at 30–33 years of age), mood episodes were assessed (n = 194). The risk of developing BPD (n = 22), compared with MDD (n = 104) or no mood episodes in adulthood (n = 68), was estimated via logistic regression. Adolescent mood symptoms, non-mood disorders, and family characteristics were assessed as potential risk factors.

**Results:**

Among the adolescents with mood disorders, a family history of BPD was the strongest predictor of developing BPD compared with having no mood episodes in adulthood (OR = 5.94; 95% CI = 1.11-31.73), whereas disruptive disorders significantly increased the risk of developing BPD compared with developing MDD (OR = 2.94; CI = 1.06-8.12). The risk that adolescents with MDD would develop adult BPD, versus having no mood episodes in adulthood, was elevated among those with an early disruptive disorder (OR = 3.62; CI = 1.09-12.07) or multiple somatic symptoms (OR = 6.60; CI = 1.70-25.67). Only disruptive disorders significantly predicted adult BPD among adolescents with MDD versus continued MDD in adulthood (OR = 3.59; CI = 1.17-10.97). Only a few adolescents with hypomania spectrum episodes continued to have BPD as adults, and anxiety disorders appeared to increase this risk.

**Conclusions:**

Although most of the identified potential risk factors are likely general predictors of continued mood disorders, disruptive disorders emerged as specific predictors of developing adult BPD among adolescents with MDD.

**Electronic supplementary material:**

The online version of this article (doi:10.1186/s12888-014-0363-z) contains supplementary material, which is available to authorized users.

## Background

Numerous studies have shown that adolescents with mood disorders are at an increased risk of continued mood disorders in early adulthood [1]-[10]. However, the presence of (hypo)manic symptoms during childhood and adolescence does not necessarily indicate a continuing course of bipolar disorder (BPD) in adulthood [11]-[14]. The early signs that predict the continued course of adolescent mood disorders are not well established. Thus, we do not know which adolescents with a mood disorders will develop BPD, major depressive disorder (MDD), or neither as adults.

BPD is a severe condition associated with substantial impairments in emotional, cognitive, and social functioning [15]-[18]. An increased knowledge regarding the early signs of BPD might provide insight regarding the development of the mood disorder and help identify individuals at risk of developing BPD and enable early intervention.

Adolescent BPD is associated with early signs such as mood lability or swings, anxiety, hyperarousal, somatic complaints, behavioral dysregulation, attention difficulties and school problems [5],[18]-[22]. Several studies have investigated whether the early signs of psychopathology predict BPD later in life. Numerous studies have demonstrated high rates of developing mania among children or adolescents with depression [23]-[28]. Therefore, early-onset depressive symptoms or MDD might predict later BPD. Disruptive behavioral disorders, in combination with mood changes, have been identified as more specific markers of the early onset of BPD [5],[27],[29]-[32]. In addition, previous authors have found that the presence of anxiety disorders, especially panic disorder, might be a marker of the early onset of BPD [7],[33]-[35].

Still, the best-established early marker of BPD risk remains family history [14],[36],[37]. This factor has been widely accepted in clinical practice, despite the fact that the majority of the high-risk offspring of individuals with mood disorders do not develop BPD [38]-[41]. However, a large proportion of offspring develop other mental disorders [42].

The clinical usefulness of the early markers/premorbid problems as predictors of subsequent BPD has not been proven. The generally high frequency of pre-morbidities and comorbidities between adolescent mood disorders, externalizing disorders and internalizing disorders raises questions regarding the relevance of these disorders for the continued disease course.

To summarize, conclusive findings within this area of research are sparse and additional research is needed. The present study is based on a unique community sample of adolescents with mood disorders, followed up after 15 years. Although previous publications form this cohort have not focused on the potential risk factors of BPD, certain results have indicated that specific factors might be important. We have shown that long-term adolescent depression strongly predicts both continued MDD and BPD in adulthood [10]. In another publication, we reported that multiple somatic symptoms in adolescence independently predict both continued MDD and BPD in adulthood [43],[44]. Depressed adolescents with more than four somatic symptoms had particularly poor outcomes, with high rates of severe, recurrent, and chronic depression or BPD. Somewhat surprisingly, we also found, that adolescents with hypomania spectrum episodes did not have a higher risk of BPD in adulthood compared with those with only MDD [14]. On the other hand, a family history of BPD appears to predict BPD in adulthood. Adolescents with either hypomania spectrum disorder or MDD, who also had a 1^st^ - and/or 2^nd^ - degree family member with BPD, were more likely to have BPD as adults compared with those without this history. Adolescents with MDD and a 1^st^ - and/or 2^nd^ - degree relative with BPD were more likely to develop BPD versus those with MDD and no such history. Similarly, adolescents with hypomania spectrum disorder tended to have (hypo)mania episode(s) in adulthood if they had a 1^st^ - and/or 2^nd^ - degree family member with BPD.

The present study includes a range of potential child and adolescent risk factors for developing BPD. Our overarching aim was to identify the early risk factors of adult BPD (compared with MDD or no mood episodes in adulthood) among individuals with adolescent mood episodes. We investigated the potential risk factors for the following:

- adult BPD among individuals with previous mood episodes (either MDD or hypomania spectrum episodes) during adolescence;
- the development of adult BPD among those with adolescent hypomania spectrum episodes; and
- the development of adult BPD among those with adolescent MDD.

## Methods

### Study design and participants

**Figure 1:**
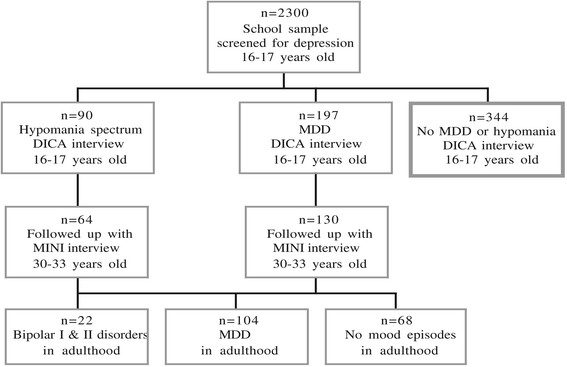
**Chart illustrating the selection of participants and division into groups for the present study.** Note: Of the 2 300 adolescents originally screened for depression, participants with positive screening and an equal number of peers with negative screening, were diagnostically interviewed.

This study examined the early risk factors for BPD in a high-risk community sample of adolescents with mood disorders. The data were prospectively collected in two waves, with a baseline assessment at age 16–17 years of age and a blinded follow-up assessment at 30–33 years of age (see Figure 1). We assessed adolescent risk factors for BPD at the follow-up assessment compared with 1) MDD in adulthood and 2) no mood episodes in adulthood.

Detailed methods of this community-based study have been published elsewhere [10],[14],[45]. Briefly, 2,300 of 2,446 (93%) 16- to 17-year-olds in a mid-sized Swedish community participated in a screening procedure aimed at identifying individuals with depressive symptoms using the Beck Depression Inventory-Child (BDI-C) [46],[47] and the Centre for Epidemiological Studies – Depression Scale for Children (CES-DC) [48]-[50]. Students with positive screenings (BDI ≥ 16, CES-DC ≥ 30 + BDI ≥ 11, or a previous suicide attempt) were interviewed using the revised form of the Diagnostic Interview for Children and Adolescents according to the DSM-III-R for adolescents (DICA-R-A) [51]. For each student with a positive screening, a same-sex classmate with a negative screening was interviewed in the same manner. In total, 631 adolescents were interviewed and invited to consent to a follow-up study.

In the present study, participants with a DICA-R-A diagnosis of MDD (n = 197), a hypomania spectrum episode (n = 90), or both at the first evaluation (i.e., 16–17 years of age) were included. A hypomania spectrum episode was defined as having an “elevated mood”, “grandiosity” or both and at least 1–3 additional manic symptoms or irritability as the only core symptom and at least 4 additional manic symptoms. The participants either met the criteria for full syndromal hypomania (n = 40) or brief-episode hypomania (less than four days of symptoms; n = 18) or subsyndromal hypomania (1 or 2 core symptoms and 1–2 additional symptoms were full-filled) (n = 32). The majority had also experienced a major depressive episode (n = 68); 10 participants had subthreshold depression and twelve had no depression.

To ensure that the symptoms were not better explained by ADHD, we determined that the 12 participants in the hypomania group without depression did not meet the criteria for ADHD.

### Procedure

At baseline, the participants were assessed using the DICA-R-A. The participants also completed numerous self-rating scales, including the Somatic Checklist Instrument (SCI) [52]. A psychiatrist conducted more than half of the interviews, while staff trained by this psychiatrist (two psychiatric nurses, two psychologists, and a student) conducted the rest of the interviews. In order to ascertain inter-rater reliability simultaneous scoring were done in 27 interviews, with only minor discrepancies between the raters.

A follow-up assessment was conducted after 15 years, when the participants were 30–33 years of age. A blinded assessment of adult mental disorders was conducted with the Mini International Neuropsychiatric Interview Plus [53]. Episodes of major depression, hypomania and mania were rated between age 19 and the follow-up assessment. To enhance participant recall of mood episodes during the investigated period, a life-chart with questions concerning life events, mood episodes, and treatments was used. In addition, information regarding family histories of mood disorders in 1^st^ - and/or 2^nd^ - degree relatives were collected at the follow-up assessment. The five interviewers, trained in clinical psychology or psychiatry, were blinded to the information from the baseline evaluation. To enhance inter-rater reliability, each interviewer was video-recorded once, and the recorded interviews were rated by all interviewers. These recordings yielded an overall free-marginal kappa value of 0.93. To further increase reliability, and to ensure the clinical validity of the diagnoses, uncertainties were regularly discussed with senior psychiatrists during sessions of group supervision.

All participants (n = 194) with an adolescent mood disorder who participated in the follow-up evaluation were included in the analyses. The participants with a diagnosis of bipolar I or II disorder in adulthood were compared with those with MDD and those with no mood episodes in adulthood with regard to potential risk factors.

### Potential risk factors

Several risk factors with potential relevance for the continued course of mood disorder were considered in the analyses, including child and adolescent mental disorders, adolescent mood symptoms, somatic symptoms, adverse life events during childhood and adolescence, and a family history of mood disorders in 1^st^ - and/or 2^nd^ - degree relatives.

Previous and current child and adolescent mental disorders as well as psychotic and affective symptoms were recorded using the DICA-R-A. The following diagnoses were included in the analyses: separation anxiety; avoidant disorder (social phobia); overanxiousness (GAD); panic disorder; obsessive-compulsive disorder (OCD); posttraumatic stress disorder (PTSD); eating disorders (i.e., anorexia nervosa and bulimia nervosa); disruptive disorders (i.e., conduct disorder [CD], oppositional defiant disorder [ODD] or attention-deficit/hyperactivity disorder [ADHD]); and substance abuse (drugs, glue or alcohol).

In addition, long-term depression among adolescents was included as a predictor in the analyses because previous analyses showed that this variable strongly predicts continued mood disorders [10]. Long-term depression was defined as major depression during most of the previous year, major depression followed by symptoms meeting the criteria for dysthymia, or major depression superimposed on a state of dysthymia.

Childhood psychotic symptoms, other than schizophrenia, concerned present or previous delusional symptoms or hallucinations that were not due to the direct physiological effects of a substance (e.g., drug abuse and medication) were included in the analyses.

The included adolescent hypomanic symptoms were elevated mood, grandiosity, irritability, distractibility, increased activity, racing thoughts, pressured speech and a decreased need for sleep. The depressive episode symptoms were suicide attempt, suicide ideation, dysphoria, anhedonia, psychomotor inhibition, fatigue, feelings of worthlessness, problems with concentration, and sleep and appetite disturbances. Details regarding the mood symptoms recorded in this cohort have been published previously [14].

The presence of multiple somatic symptoms was included as a potential risk factor because previous analyses have shown that these symptoms predict continued mood episodes [43],[44]. The SCI assesses various physical symptoms via 22 items [54]. Each symptom was graded with regard to frequency (0 = never, 1 = monthly, 2 = weekly, 3 = several times a week, and 4 = daily) and intensity (0 = no problem, 1 = minor, 2 = moderate, 3 = troublesome, and 4 = extremely troublesome). The present study categorized a somatic symptom as such when its frequency and intensity were multiplied, and a score ≥6 was obtained (e.g., 2 × 3: weekly and troublesome symptoms). This categorization excluded the possibility that monthly premenstrual symptoms were recorded as somatic symptoms. Multiple somatic symptoms were defined as 5 or more symptoms.

The DICA-R-A [51] also included questions regarding adverse life events during adolescence. Items regarding family histories of substance abuse, family violence and deaths in the family due to an accident were selected. Details of the baseline characteristics have been published previously [49],[55].

At the follow-up assessment, the participants reported their family histories of depressive episodes or manic/hypomanic episodes among their 1^st^ - or 2^nd^ - degree relatives (i.e., parents, siblings, children, grandparents, uncles, aunts, nephews, nieces, and half-siblings).

### Antidepressant treatment in childhood, adolescence and adulthood

Antidepressant medication has been reported to trigger (hypo)manic episodes in some patients. We used information from both the baseline assessment and the follow-up interview in order to ascertain that the (hypo)manic episodes reported were not attributable to medication: Treatments during childhood and adolescence were assessed with the DICA-R-A, and no participant reported psychotropic medication. At follow-up, the MINI interview was used to identify mood episodes and a life-chart was used to indicate when mood episodes had occurred and when the individual had received treatment for the mood episodes. Of the 22 participants reporting bipolar disorder in adulthood, 14 reported treatment with antidepressants. However, all these 14 participants reported that the first (hypo)manic episode had occurred before the antidepressant treatment was introduced.

### Follow-up attrition

The participation rate at the follow-up evaluation was 66% (130/197) among those with adolescent MDD and 71% (64/90) among those with adolescent hypomania spectrum episodes. No major differences were identified between those who participated and those who were lost to follow up. Details regarding the follow-up attrition have been published previously [45].

### Statistical analyses

In the first set of analyses, adolescents with MDD or hypomania spectrum episodes were divided into three groups: those who developed BPD in adulthood; those who developed MDD in adulthood; or those who did not developed mood episodes in adulthood (Table 1). Differences in risk factors (previous diagnoses, clinical characteristics and family characteristics) were analyzed using a univariate logistic regression. In the second step, statistically significant risk factors were entered as covariates into multivariate logistic regression models. In the first model, the outcome variable was BPD versus no mood episodes in adulthood. In the second model, the outcome variable was BPD versus MDD in adulthood.

**Table 1:** **Potential child and adolescent risk factors of adult bipolar disorder (BPD) compared with major depression disorder (MDD) or no mood episodes in adulthood**

Potential risk factors in childhood/adolescence	A	B	C	A vs. C	A vs. B
	BPD in adulthood	MDD in adulthood	No mood episode in adulthood	OR (95% CI)	OR (95% CI)
	n = 22 (%)	n = 104 (%)	n = 68 (%)		
Female	19 (86)	87 (84)	51 (75)	2.11 (0.56-8.03)	1.24 (0.33-4.65)
**Mental disorders (DICA-R-A):**					
Separation Anxiety disorder	10 (46)	40 (39)	20 (29)	2.00 (0.75-5.37)	1.33 (0.53-3.37)
Social Phobia	2 (9)	17 (16)	9 (13)	0.66 (0.13-3.29)	0.51 (0.11-2.40)
GAD	8 (36)	47 (45)	14 (21)	2.20 (0.77-6.29)	0.69 (0.27-1.79)
Panic Disorder	5 (23)	16 (15)	5 (7)	3.71 (0.96-14.30)	1.62 (0.52-5.01)
Any anxiety disorder^a^	15 (68)	71 (68)	35 (52)	2.02 (0.73-5.58)	1.00 (0.37-2.67)
OCD	4 (18)	27 (26)	12 (18)	1.04 (0.30-3.62)	0.63 (0.20-2.04)
PTSD	-	6 (6)	-	-	-
Eating disorders	2 (9)	5 (5)	4 (6)	1.60 (0.27-9.39)	1.98 (0.36-10.93)
Disruptive disorder (CD/ODD/ADHD)	13 (59)	30 (29)	20 (29)	3.47 (1.28-9.40)*	3.56 (1.38-9.21)**
Substance abuse	3 (14)	10 (10)	8 (12)	1.18 (0.29-4.92)	1.47 (0.37-5.85)
**Mania episode symptoms:**					
Elevated mood	5 (23)	34 (33)	25 (37)	0.51 (0.17-1.54)	0.61 (0.21-1.78)
Grandiosity	7 (32)	35 (34)	29 (43)	0.63 (0.23-1.74)	0.92 (0.34-2.46)
Irritability	2 (9)	7 (7)	4 (6)	1.60 (0.27-9.39)	1.39 (0.27-7.17)
Distractibility	1 (5)	14 (14)	9 (13)	0.31 (0.04-2.61)	0.31 (0.04-2.46)
Increased activity	6 (27)	31 (30)	19 (28)	0.97 (0.33-2.84)	0.88 (0.32-2.47)
Racing thoughts	4 (18)	18 (17)	9 (13)	1.46 (0.40-5.30)	1.06 (0.32-3.51)
Pressured speech	3 (14)	21 (20)	12 (18)	0.74 (0.19-2.89)	0.62 (0.17-2.31)
Decreased need of sleep	5 (23)	33 (32)	23 (34)	0.58 (0.19-1.76)	0.63 (0.22-1.86)
**Depressive episode symptoms:**					
Suicide attempt	4 (18)	25 (24)	15 (22)	0.79 (0.23-2.68)	0.70 (0.22-2.27)
Suicide ideation	15 (68)	63 (61)	35 (51)	1.55 (0.57-4.18)	1.37 (0.53-3.50)
Dysphoria	20 (91)	99 (95)	63 (93)	0.79 (0.14-4.41)	0.51 (0.91-2.79)
Anhedonia	17 (77)	80 (77)	41 (60)	2.24 (0.74-6.79)	1.02 (0.34-3.05)
Psychomotor inhibition	18 (82)	79 (76)	49 (72)	1.75 (0.52-5.83)	1.42 (0.44-4.60)
Fatigue	15 (68)	82 (79)	47 (69)	0.96 (0.34-2.69)	0.58 (0.21-1.58)
Worthlessness	20 (91)	84 (81)	43 (63)	5.81 (1.25-26.98)*	2.38 (0.51-11.03)
Trouble concentration	18 (82)	82 (79)	53 (78)	1.27 (0.37-4.34)	1.21 (0.37-3.93)
Sleep disturbances	12 (55)	52 (50)	32 (47)	1.35 (0.51-3.54)	1.20 (0.48-3.02)
Appetite disturbances	18 (82)	83 (80)	49 (72)	1.75 (0.52-5.83)	1.14 (0.35-3.72)
Psychotic symptoms	2 (9)	6 (6)	3 (4)	2.17 (0.34-13.89)	1.63 (0.31-8.69)
Somatic symptoms (≥5)^b^	11 (50)	30 (31)	11 (17)	4.82 (1.67-13.88)**	2.27 (0.89-5.80)
Long-term depression^c^	14 (64)	54 (52)	23 (34)	3.42 (1.26-9.34)*	1.62 (0.63-4.19)
**Family characteristics:**					
Substance abuse in family	3 (14)	12 (12)	5 (7)	1.99 (0.44-9.10)	1.20 (0.31-4.66)
Violence in family	7 (32)	19 (18)	15 (22)	1.65 (0.57-4.78)	2.06 (0.74-5.76)
Death in family by accident	4 (18)	23 (22)	13 (19)	0.94 (0.27-3.25)	0.77 (0.24-2.51)
Family history of BPD^d^	5 (23)	8 (8)	3 (4)	6.37 (1.38-29.36)*	3.53 (1.03-12.08)*
Family history of MDD^d^	15 (68)	71 (68)	25 (37)	3.69 (1.32-10.27)*	0.99 (0.37-2.67)
Family history of BPD or MDD^d^	16 (73)	71 (68)	27 (40)	4.05 (1.41-11.65)**	1.24 (0.44-3.46)

^a^Separation Anxiety disorder, Social Phobia, GAD, or Panic Disorder.

^b^The number of individuals who completed the MINI interview and the SCL for somatic symptoms differed slightly (In the MDD group there are missing data for six persons and in the no-mood episode group for four persons).

^c^Long-term depression was defined as major depression during most of the previous year, major depression followed by symptoms meeting the criteria for dysthymia, or major depression superimposed on a state of dysthymia.

^d^1^st^ and/or 2^nd^ degree family history of BPD and/or MDD.

MDD: Major depressive disorder; OCD: Obsessive-compulsive disorder; PTSD: Posttraumatic stress disorder; ADHD: Attention-deficit/hyperactivity disorder; CD: Conduct disorder; ODD: Oppositional defiant disorder; GAD: Generalized anxiety disorder. Note: *p < 0.05; **p < 0.01.

The risk factors that differed significantly between those who developed adult BPD and those who did not have mood episodes in adulthood were used to calculate a receiver operating characteristic (ROC) curve to evaluate the sensitivity and specificity for numerous risk factors. The calculation of a ROC curve for the risk factors of BPD versus MDD in adulthood was not possible because of the low number of significant risk factors.

All analyses were first adjustment for sex. This adjustment did not change the results and was not included in the final analyses presented in the results section. In the second set of analyses, univariate logistic regressions were used to identify the risk factors for adult BPD separately for the adolescents with MDD and the adolescents with hypomania spectrum episodes. Multivariate analyses were not conducted because of the smaller sample sizes of these groups. P-values below 0.05 were considered statistically significant for all statistical analyses. IBM SPSS Statistics version 22.0 for Macintosh was used.

### Ethics

The Regional Ethical Vetting Board of Uppsala, Sweden approved this study, which was conducted in accordance with the ethical standards established in the Declaration of Helsinki. Both written and verbal information about the study was offered to the students (16–17 years). Informed consent to be contacted for a future follow-up evaluation was also collected. At age 30–33 years, the participants who had given their consent to be contacted were sent written information about the follow-up evaluation. Before consenting to take part, the participants were also informed about the study verbally via telephone. The Regional Ethical Vetting Board approved the verbal consent procedure used in the study.

## Results

### Risk factors for adult BPD among all adolescents with mood disorders

Of the 194 participants with adolescent mood disorders who were followed up after 15 years, 22 were diagnosed with bipolar I or II, 104 had MDD and 68 had no mood episodes in adulthood. The results of the univariate logistic regression analyses of the risk factors for BPD in adulthood (versus having MDD or no mood episodes) are presented in Table 1. Disruptive disorders significantly increased the risk of BPD compared with MDD (OR = 3.56; 95% CI = 1.38-9.21) and no mood episodes (OR = 3.47; CI = 1.28-9.40). In addition 1^st^ - and/or 2^nd^ - degree family histories of BPD significantly increased the risk of adult BPD compared with having MDD (OR = 3.53; CI = 1.03-12.08) or no mood episodes in adulthood (OR = 6.37; CI = 1.38-29.36).

The feeling of worthlessness was the single affective symptom from the DICA-interview that significantly increased the risk of BPD compared with not having a mood episode. The other significant risk factors for adult BPD (compared with no mood episodes in adulthood) included multiple somatic symptoms (OR = 4.82; CI = 1.67-13.88), and long-term depression (OR = 4.38; CI = 1.39-13.80). A history of child and adolescent panic disorder was not a significant risk factor (OR = 3.71; CI = 0.96-14.30). Similarly, a history of any anxiety disorder (Separation Anxiety disorder, Social Phobia, GAD, or Panic Disorder) in childhood and adolescence did not reach statistical significance as a risk factor for adult bipolar disorder compared with having no mood episodes (OR = 2.02; CI = 0.73-5.58) or MDD (OR = 1.00; CI = 0.37-2.67) in adulthood.

**Figure 2:**
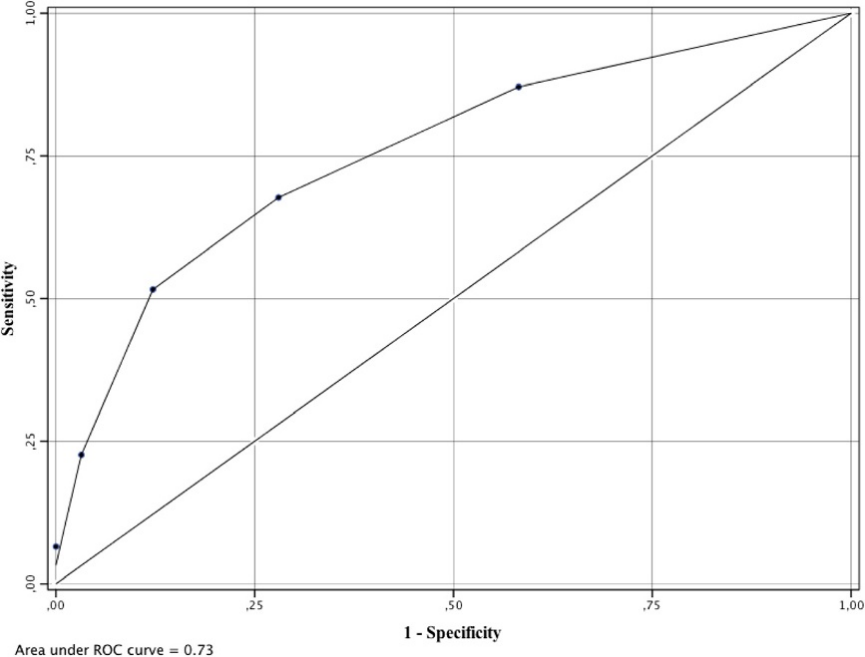
**The receiver operating characteristic (ROC) curve of adult bipolar disorder (compared with no mood episodes in adulthood) among adolescents with mood disorders, according to the number of five independent child and adolescent risk factors.** The following risk factors were included: disruptive disorders; feelings of worthlessness; multiple somatic symptoms; long-term depression; 1st and/or 2nd degree family histories of bipolar disorder. The presence of at least two risk factors resulted in a sensitivity of 68% and specificity of 72%, whereas the presence of three or more risk factors resulted in sensitivity of 52% and specificity of 88%.

The following independent risk factors were entered in multivariate logistic regression analyses: disruptive disorders; feelings of worthlessness; multiple somatic symptoms; long-term depression; and 1^st^ - and/or 2^nd^ - degree family histories of BPD. In this model, three risk factors remained significant for adult BPD compared with no mood episodes: feelings of worthlessness (OR = 5.20; CI = 1.01-27.08); 1^st^ - and/or 2^nd^ - degree family histories of BPD (OR = 5.94; CI = 1.11-31.73); and multiple somatic symptoms (OR = 3.33; CI = 1.04-10.72]). The same five risk factors for adult BPD (compared with no mood episodes) were evaluated using an ROC curve (Figure 2). The presence of at least two risk factors resulted in a sensitivity of 68% and specificity of 72%, whereas the presence of three or more risk factors resulted in sensitivity of 52% and specificity of 88%.

Only disruptive disorders significantly increased the risk of BPD compared with MDD in a multivariate analysis using the same five risk factors (OR = 2.94; CI = 1.06-8.12).

### Risk factors for adult BPD among adolescents with hypomania spectrum episodes

Of the 64 adolescents with hypomania spectrum episodes during childhood, 6 had developed adult hypomania or mania, 32 developed MDD and 26 reported no mood episodes in adulthood.

**Figure 3:**
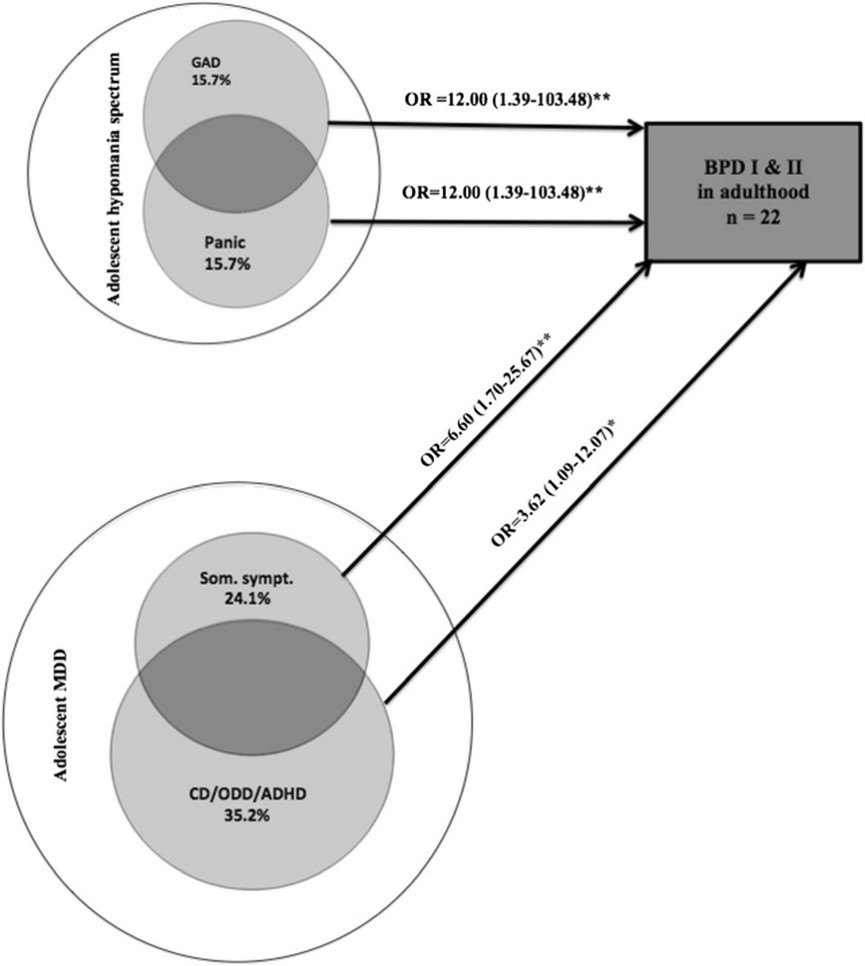
**Child and adolescent risk factors for developing bipolar disorder (BPD; n = 22) compared with no mood episodes (n = 68) in adulthood among adolescents with hypomania spectrum episodes (n = 32; 6 developed adult BPD) or transition from adolescent MDD to adult BPD (n = 58; 16 developed adult BPD).** Note: *p < 0.05; **p < 0.01.

The continuity between adolescent hypomania spectrum and adult BPD (compared with no mood episode) was associated with panic disorder (OR = 12.00; CI = 1.39-103.48), GAD (OR = 12.00; CI = 1.39-103.48) and long-term depression (OR = 12.00; CI = 1.39-103.48). When these three factors were entered simultaneously into a logistic regression analysis, panic disorder and GAD predicted an increased risk of adult BPD, whereas long-term depression did not remain as significant (Figure 3). A trend was also observed for an increased risk of continued adult BPD (compared with having no mood disorder) with regard to the presence of 1^st^ - and/or 2^nd^ - degree family histories of BPD (OR = 12.50; CI = 0.91-172.08) and the 1^st^ - and/or 2^nd^ - degree family histories of MDD (OR = 9.44; CI = 0.95-93.64).

**Figure 4:**
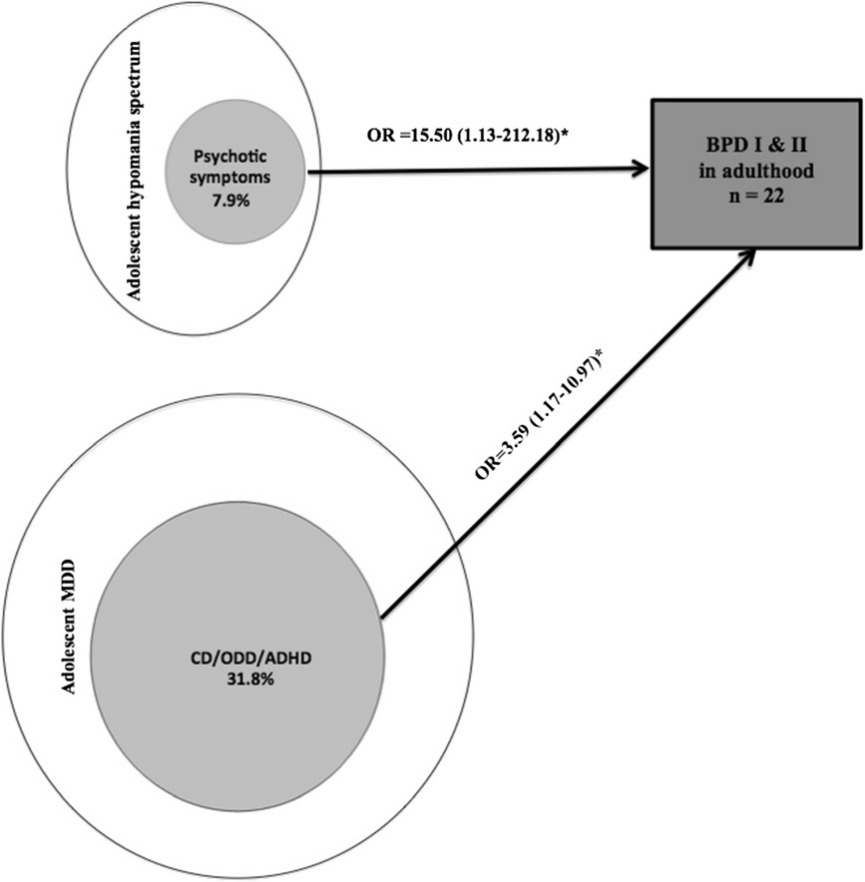
**Child and adolescent risk factors for developing bipolar disorder (BPD; n = 22) compared with major depressive disorder (MDD; n = 104) in adulthood among adolescents with hypomania spectrum episodes (n = 38; 6 developed adult BPD) or transition from adolescent MDD to adult BPD (n = 88; 16 developed adult BPD).** Note: *p < 0.05.

Continuity between adolescent hypomania spectrum and adult BPD (compared with MDD in adulthood) was associated with psychotic symptoms in adolescence (OR = 15.50; CI = 1.13-212.18; Figure 4).

### Risk factors for developing adult BPD among adolescents with MDD

Out of the 130 adolescents with MDD during adolescence, 72 developed adult MDD, 16 developed hypomania or mania and 42 reported no mood episodes in adulthood. The transition from adolescent MDD to adult BPD (compared with no mood episodes in adulthood) was associated with the presence of disruptive disorders (OR = 3.62; CI = 1.09-12.07) and multiple somatic symptoms (OR = 6.60; CI = 1.70-25.67; Figure 3). A trend was observed for the increased risk of BPD with regard to 1^st^ -and/or 2^nd^ - degree family histories of BPD or MDD (OR = 3.24; CI = 0.95-11.00).

The transition from adolescent MDD to adult BPD (compared with continuing MDD in adulthood) was only significantly associated with adolescent disruptive disorders (OR = 3.59; CI = 1.17-10.97; Figure 4).

## Discussion

This study analyzed unique data from a prospective 15-year follow-up community sample of adolescents with mood disorders to identify the early risk factors for adult BPD. Numerous child and adolescent factors differed between those who developed BPD and those who did not have any mood episodes in adulthood, including family histories of BPD, multiple somatic symptoms, and anxiety disorders. Disruptive disorder in childhood or adolescence as well as family histories of BPD emerged as significant risk factors that differentiated between the future development of BPD and MDD. However, no predictor clearly delineated the group of adolescents who subsequently developed BPD as adults.

Our results are in line with previous studies showing that a family history of BPD is a robust risk factor for this disorder [23],[27],[36],[37],[56]-[61]. This finding is also consistent with genetic-epidemiological and genome–wide linkage studies [62]-[64]. However, specific predictors have been difficult to determine within high-risk offspring populations [25],[27],[40],[58],[65],[66]. This difficulty might be partially explained by the fact that previous studies have not differentiated between the continuity of (hypo)mania symptoms during childhood and adolescence into adult BPD or the transition from adolescent MDD to adult BPD. In addition, the genetic heterogeneity of BPD might influence its different trajectories; therefore, different subtypes of BPD might exist and further differentiation studies are needed.

Our results are also in keeping with previous studies suggesting that anxiety disorders are significant predictors of bipolar spectrum disorders. These studies indicate that anxiety disorders precede the onset of BPDs in general [7],[40],[67]-[71], or frequently overlap with BPDs [72]-[74]. Our results highlight panic disorder and GAD as potential risk factors for continued BPD among adolescents with hypomania spectrum. Several studies have suggested that panic disorder shares genetic and family histories with BPD [34],[35],[75],[76]. Panic disorder is often associated with BPD rapid cycling [77], as are disruptive disorders [78],[79].

The presence of a disruptive disorder was the only factor associated with the transition from adolescent MDD to adult BPD (compared with continuation of MDD). Several longitudinal studies have found an association between early disruptive disorders and the bipolar spectrum [30],[31],[40],[80]. Both disruptive disorders and BPD are associated with a low threshold for arousal and related to high emotional reactivity. It is possible that disruptive behavioral features and bipolar symptoms may be part of a continuum, and disruptive behavioral symptoms may serve as an early marker of BPD [5],[20],[30],[81],[82]. However, a previous high-risk offspring study concluded that behavioral disorders are not a specific predictor of BPD; rather the risk of having this condition seems to be elevated among subgroups of offspring with parents with BPD who did not respond to lithium [27],[40],[65]. It is possible that disruptive disorders are not only comorbid to BPD, but might also precede bipolar disorders or even represent an early stage of the disorder.

Different types of risk factors predominated among adolescents with MDD who developed adult BPD and those with hypomania spectrum episodes who developed adult BPD. Disruptive disorders, multiple somatic symptoms (somatic complaints), or both precede adolescent MDD before developing adult BPD. Furthermore, panic disorder, GAD or both were associated with adolescent hypomania before developing adult BPD. It is possible that these two predictor groups might represent different developmental trajectories of adult BPD. The first subtype, which is preceded by disruptive disorders, somatic symptoms (somatic complaints) or both, appears to overlap with characteristics of irritability, explosive anger, aggression, mood lability and somatic complaints, which are criteria for the cyclothymic temperament [2],[83],[84]. Furthermore, unstable personality features such as cyclothymic temperament and borderline personality disorder, are more common among patients with BPD than among patients with unipolar depression [84]-[90]. On the other hand, the second subtype, represented by anxiety disorders, might be best viewed as an expression of a general vulnerability factor for mood disorders rather than a bipolar-specific risk factor. Our findings also show that anxiety disorders can precede adult BPD, which is in line with previous studies [2],[35],[58],[59],[91].

Psychotic symptoms might play a similar role. Among adolescents with a hypomania spectrum disorder, early psychotic symptoms appeared to increase the risk of BPD in adulthood. However, psychotic symptoms only occurred in a few individuals, and these results should be interpreted with caution. Early psychotic symptoms that lead to later mood disorders are more clearly aligned with BPD than unipolar depressive disorders [91]-[93]. This association is also in line with previous studies emphasizing the role of the psychotic features related to early onset mood disorder and the increased risk of BPDs or schizoaffective disorder in long-term follow-up studies [94]-[100].

Thus, psychotic symptoms in childhood and adolescence probably have diagnostic and prognostic values. Progressive brain changes might occur after the first psychotic/depressive episode, resulting in the inadequate maturation of the cortex [101]-[108] and provoking mild but progressive brain dysfunction with cognitive impairments in participants with bipolar disorder [103],[109]-[112]. More research is needed on this topic.

### Clinical considerations

Although no predictor of high sensitivity or specificity was identified, the results may still have important implications for clinical practice. In the present study, 11.3% of the participants developed adult BPD. This rate was substantially higher in subsamples with the major identified risk factors, including 31.3% of participants with family histories of BPD, 20.6% of participants with disruptive disorders, and 19.2% of participants with panic disorder. However, none of the studied predictors had specificity or sensitivity values high enough to be used in routine clinical practice with regard to informing patients and their families of the supposed long-term course of the disorder or facilitating decisions on the long-term use of mood stabilizers. However, given the serious nature of BPD and recurrent MDD, adolescents with mood disorders and, in particular, those with early indications of an increased risk for future BPD should be followed up with carefully, preferably in specialized affective disorder subunits, where the long-term results are best [113],[114].

### Strengths and limitations

This study has numerous strengths. The study was based on a large, well-defined community sample, and it focused on the natural development of mood disorders. Participants were assessed during adolescence and followed up 15 years later by clinically trained interviewers blinded to the adolescent interviews. We had access to extensive data about mood episodes, other mental disorders, and treatment. Of note, no case of suspected antidepressant induced switch was identified in this sample, although we had data on the timing of mood episodes and antidepressant treatment.

Certain limitations of this study must also be considered. First, the participants lost to follow-up might have more severe mood disorders. However, separate analyses demonstrated that those lost to follow-up and those who participated did not differ substantially with regard to adolescent psychopathology [14],[45],[115]. Furthermore, the complete sample has been followed in Swedish national registers data [116]. The registers indicated that only few individuals had been treated for BPD, which suggests that we did not miss many severe cases.

Second, although we included both adolescents with MDD and hypomania spectrum episodes, the original community study was designed to screen for only depression and not for hypomania. No hypomania screening measurement was used at the baseline evaluation, and therefore some individuals with ongoing hypomania might not have been identified. A fully representative sample of adolescents with hypomania spectrum episodes might show a slightly different result. However, 317 controls with negative screening results were also diagnostically interviewed. In addition, numerous studies have demonstrated that the early onset of depressive disorders in children or adolescents typically precedes BPD [3],[23],[24],[27],[40],[117],[118]. It should also be noted that no hypomania rating scale was used at follow-up. Although all participants at this stage were diagnostically interviewed, a rating scale could have added a dimensional perspective.

Third, there are some potential limitations regarding to the validity of our data. All information was self-reported. Further, we did not include potential predictors such as socioeconomic status and gender. Finally, a general risk of type II errors exists, because of the relatively small number of participants. Larger samples are needed in future studies.

## Conclusion

The current results provide an overview of a range of potential clinical risk factors of adult BPD among adolescents with mood disorders. No risk factor of high sensitivity or specificity was identified. Because of the severity of BPD, however, adolescents with mood disorders should be followed carefully into adulthood. Characteristics such as family histories, disruptive disorders, anxiety disorders, somatic symptoms, and family histories of mood disorders warrant particular attention. In order to make progress, it is likely that future studies need to include larger samples and account for both genetic factors and psychosocial exposure during critical periods.

## Authors’ original submitted files for images

Below are the links to the authors’ original submitted files for images. Authors’ original file for figure 1 Authors’ original file for figure 2 Authors’ original file for figure 3 Authors’ original file for figure 4

## Abbreviations

DSM-IV: American Psychiatric Association Diagnostic and Statistical Manual of Mental Disorders
DICA: Diagnostic Interview for Children and Adolescents in the Revised form According to DSM-III-R for Adolescents
BDI-C: Beck Depression Inventory-Child
CES-DC: Centre for Epidemiological Studies – Depression Scale for Children
MINI: Mini International Neuropsychiatric Interview Plus
BPD: Bipolar disorder
SCI: The Somatic Checklist Instrument
MDD: Major depression disorder
OCD: Obsessive-compulsive disorder
ADHD: Attention-deficit/hyperactivity disorder
CD: Conduct disorder
ODD: Oppositional defiant disorder
PTSD: Posttraumatic stress disorder
GAD: Overanxiousness
ROC curve: Receiver operating characteristic curve
