# Supplementary figures and images for: Early risk factors for adult bipolar disorder in adolescents with mood disorders: a 15-year follow-up of a community sample
Source: BMC Psychiatry. 2014 Dec 24;14:363. doi: 10.1186/s12888-014-0363-z (PMC4299780; doi:10.1186/s12888-014-0363-z)

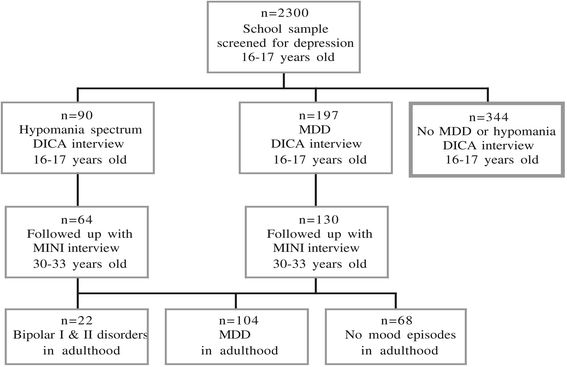

Supplement: Supplementary file 1 — Authors’ original file for figure 1 [file 12888_2014_363_MOESM1_ESM.gif]

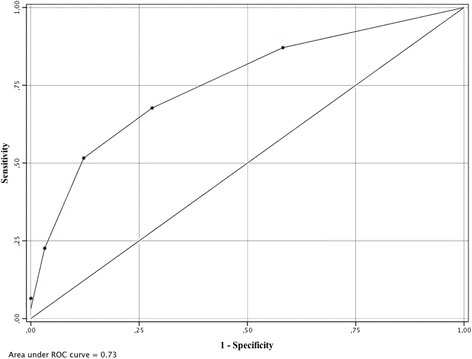

Supplement: Supplementary file 2 — Authors’ original file for figure 2 [file 12888_2014_363_MOESM2_ESM.gif]

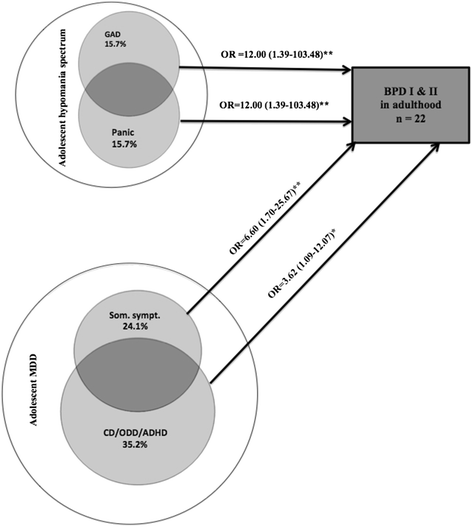

Supplement: Supplementary file 3 — Authors’ original file for figure 3 [file 12888_2014_363_MOESM3_ESM.gif]

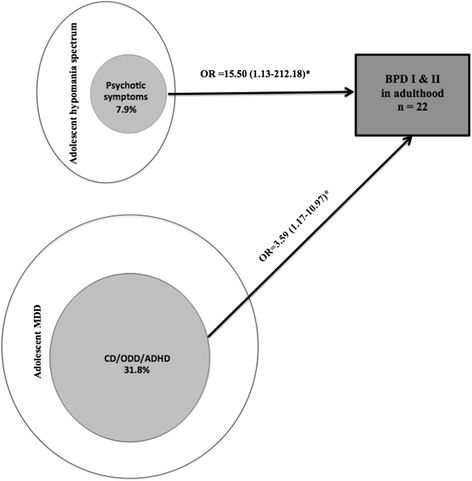

Supplement: Supplementary file 4 — Authors’ original file for figure 4 [file 12888_2014_363_MOESM4_ESM.gif]
